# Supplementary material for: Splice-Junction-Based Mapping of Alternative Isoforms in the Human Proteome
Source: Cell Rep. Author manuscript; Available in PMC 2020 Jan 15. (PMC6961840; doi:10.1016/j.celrep.2019.11.026)
Supplement: 2 [file NIHMS1546469-supplement-2.zip › DF1/PXD006675/MitralValve/MitralValve_15_PRDX5_AAAMAPIKTHLPGFVEQAEALK.pdf]

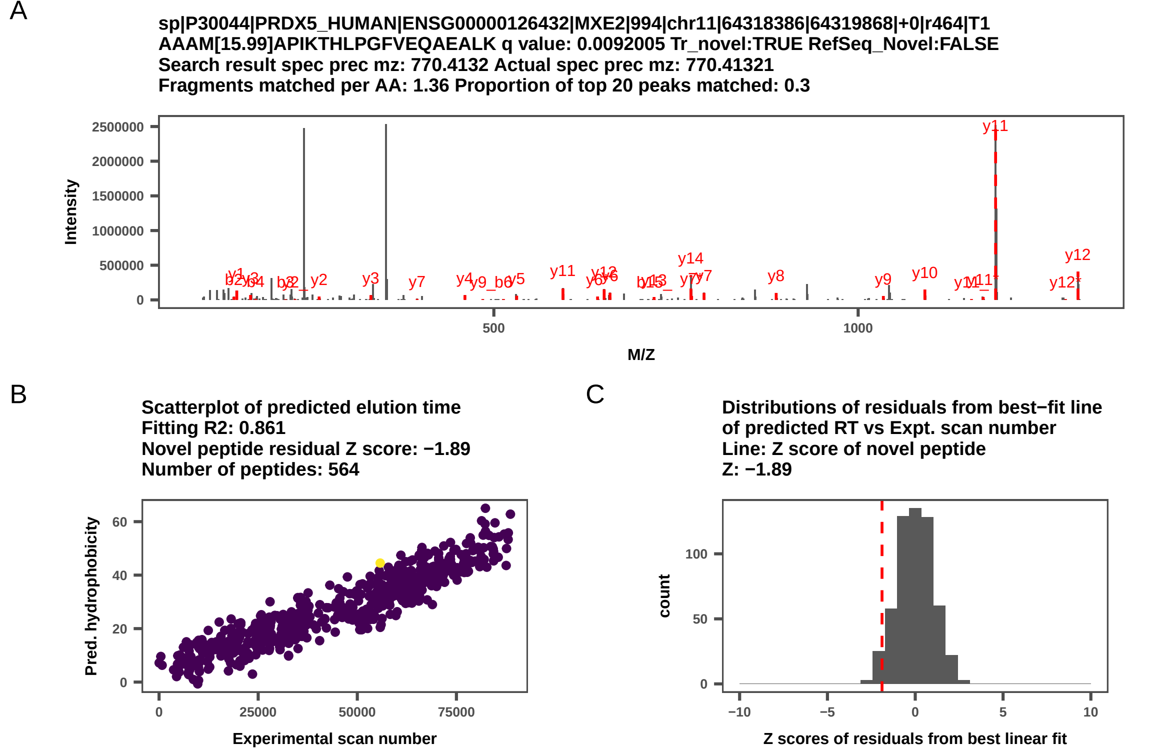
A
sp|P30044|PRDX5_HUMAN|ENSG00000126432|MXE2|994|chr11|64318386|64319868|+0|r464|T1
AAAM[15.99]APIKTHLPGFVEQAEALK q value: 0.0092005 Tr_novel:TRUE RefSeq_Novel:FALSE
Search result spec prec mz: 770.4132 Actual spec prec mz: 770.41321
Fragments matched per AA: 1.36 Proportion of top 20 peaks matched: 0.3
Intensity
2500000
2000000
1500000
1000000
500000
0
M/Z
500
1000
y11
y12
y14
y10
y9
y8
y3
y2
y7
y4
y5
B
Scatterplot of predicted elution time
Fitting R2: 0.861
Novel peptide residual Z score: −1.89
Number of peptides: 564
Pred. hydrophobicity
60
40
20
0
Experimental scan number
0
25000
50000
75000
C
Distributions of residuals from best−fit line
of predicted RT vs Expt. scan number
Line: Z score of novel peptide
Z: −1.89
count
100
50
0
Z scores of residuals from best linear fit
−10
−5
0
5
10
